# Supplementary material for: Origin of heavy rare earth mineralization in South China
Source: Nat Commun. 2017 Feb 21;8:14598. doi: 10.1038/ncomms14598 (PMC5321793; doi:10.1038/ncomms14598)
Supplement: Supplementary Information — Supplementary Figures and Supplementary Tables [file ncomms14598-s1.pdf]

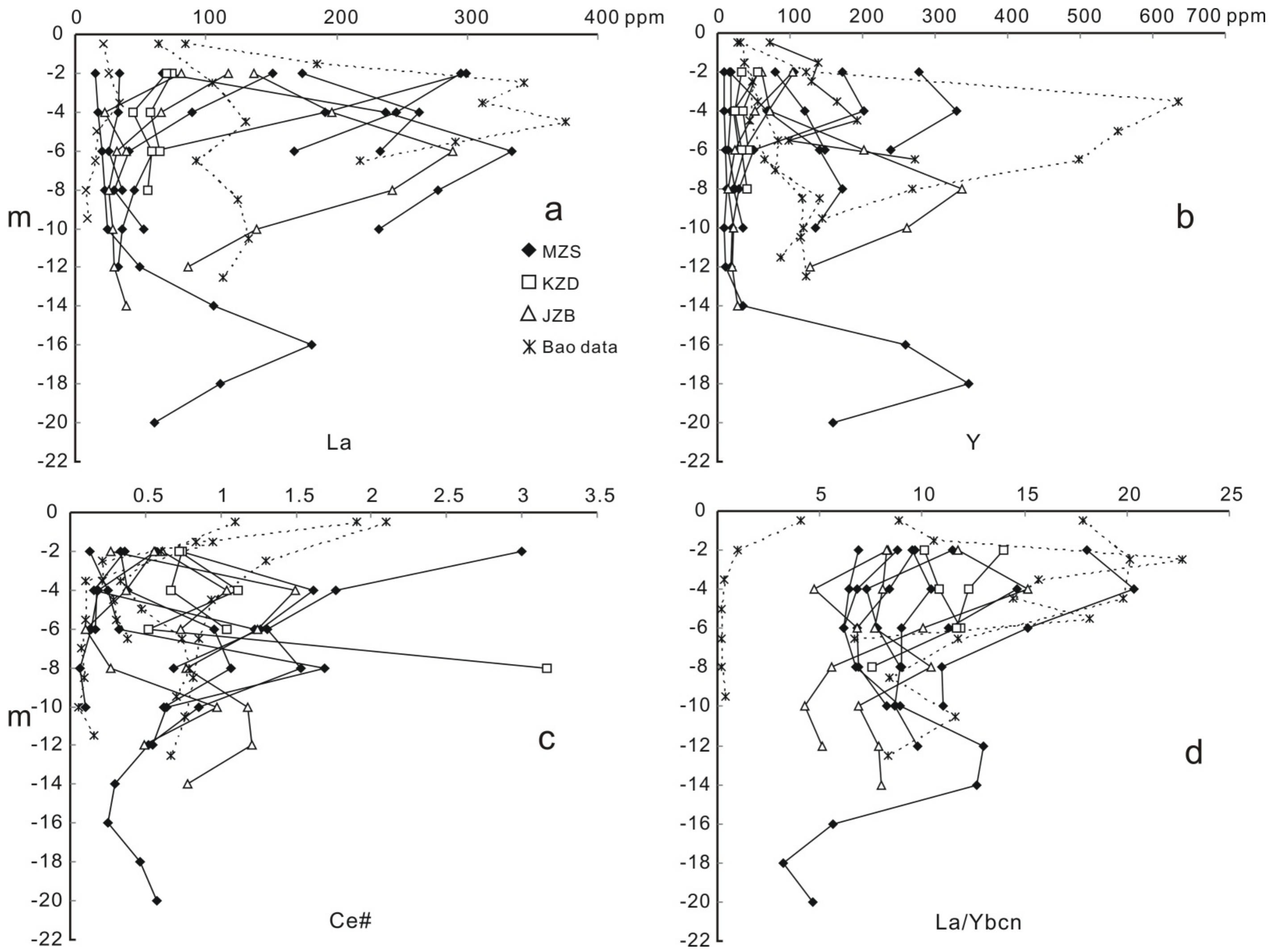

Supplementary Figure 1 | Compositional change of REE of weathering profiles with increasing drilling depth. (a) and (b) use La and Y as proxies for total light and heavy REE, respectively. (c) Ce# is chondrite normalized Ce anomaly: $Ce_{CN}/(La_{CN}*Pr_{CN})^{1/2}$. (d) La/Yb_{CN} is chondrite normalized ratio. Weathering profiles from MZS (Muzishan), KZD (Kaizidong), JZB (Jiazibei) were collected in the Zhaibei area. Previous data are from weathering profiles in the Xinxiu, Guposhan, Huashan and Heling areas[14].

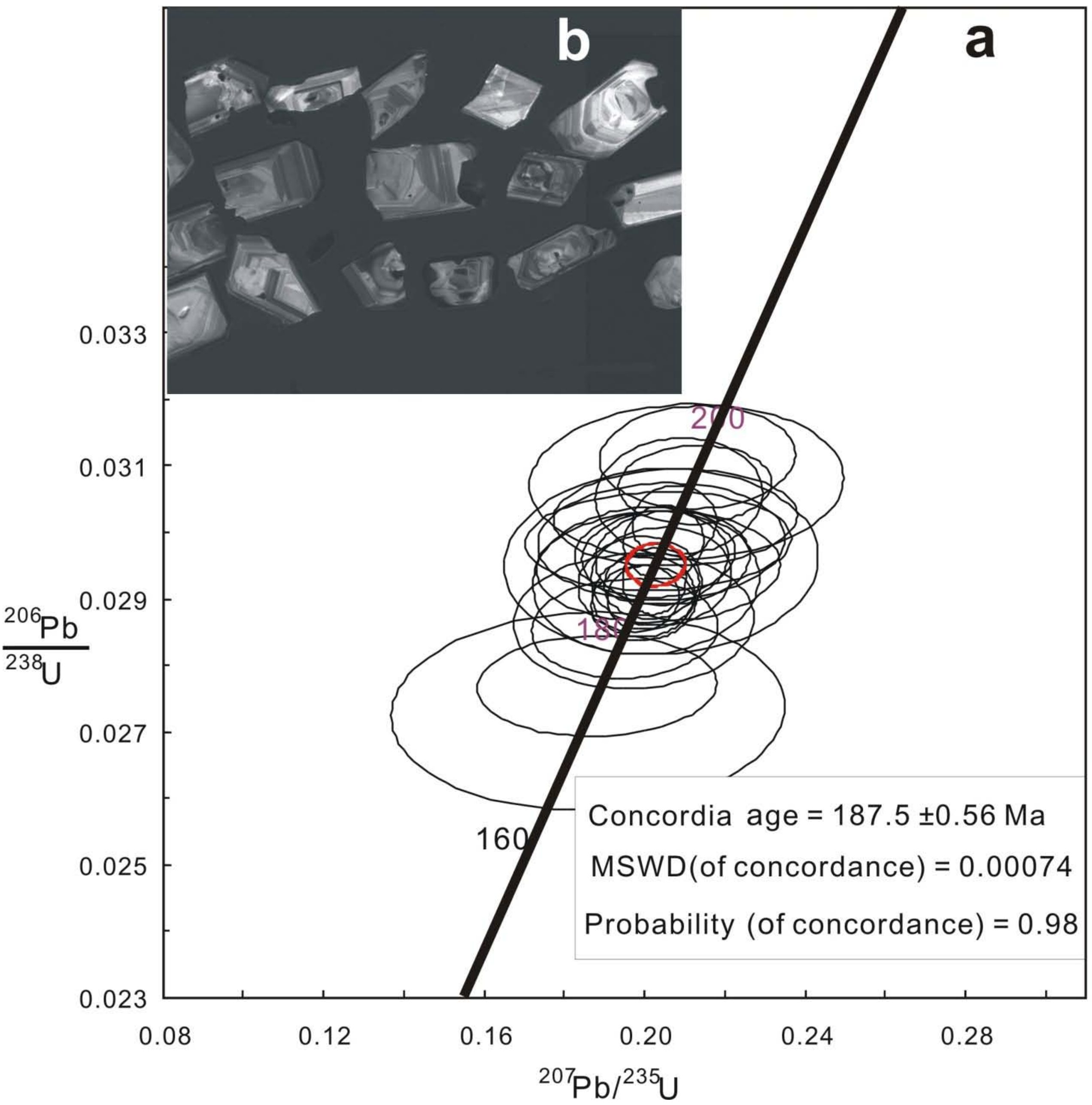

Supplementary Figure 2 | Concordia plots of LA-ICP-MS U-Pb results used in calculating the zircon age of the granites. (a) the granite formation at 188 Ma. (b) zircon cathodoluminescence image (field of 14 view: 650 μm).

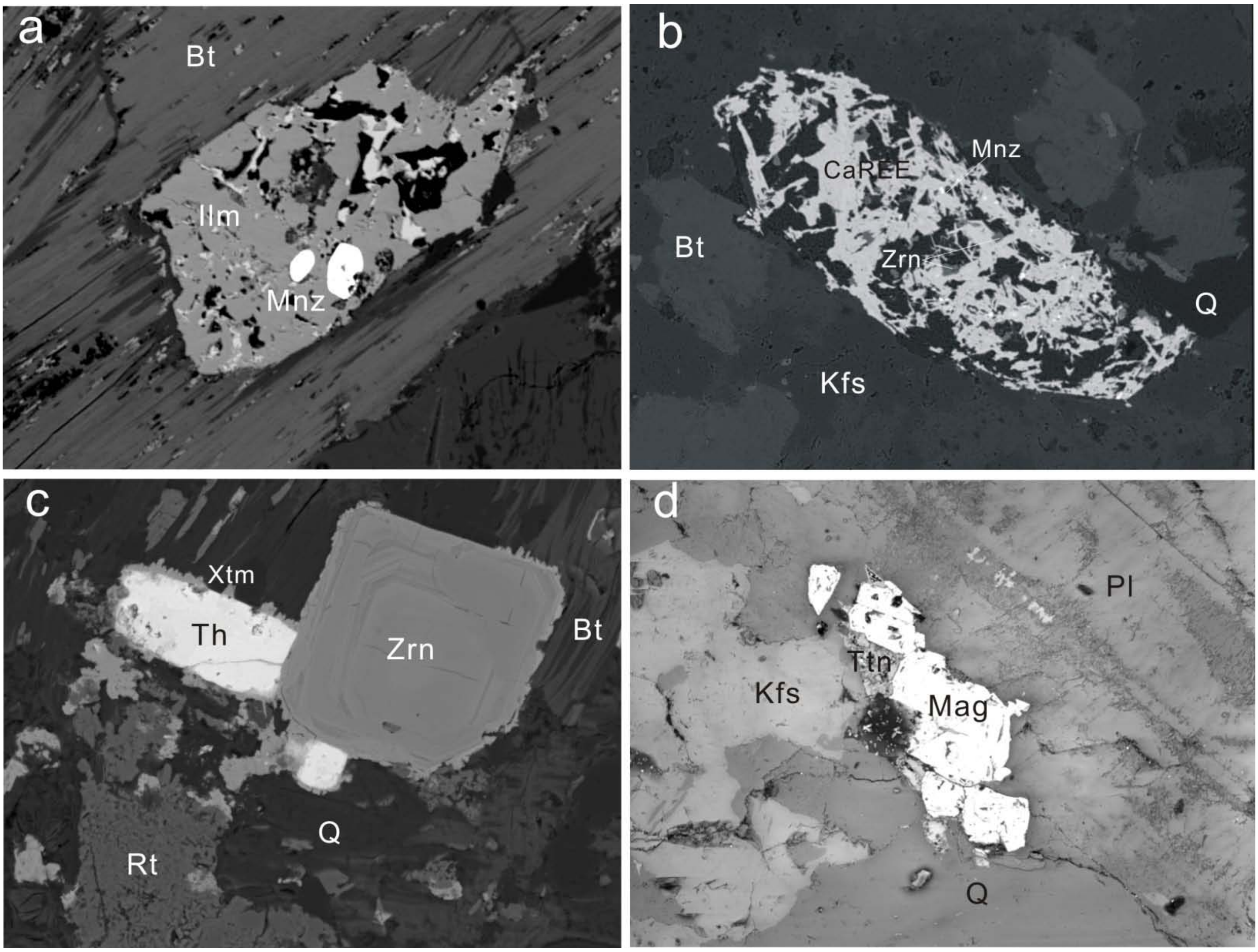

Supplementary Figure 3 | Selected characteristic textures observed in the granites

(a) primary monazite-(Ce) in ilmenite.

(b) secondary Ca-REE fluorcarbonates inter-grown with zircon and rutile, showing a pseudomorph of an earlier REE-Zr-Ti mineral (mosandrite?). (c) xenotime-(Y) occurring as radiating spines and densely packed aggregates around zircon and thorite. (d) titanite+magnetite+quartz assemblage used to calculate oxygen fugacity of the early granitite magmas. Ilm, ilmenite; Mnz, monazite-(Ce); Bt, biotite; Kfs, K-feldspar; CaREE, Ca-REE fluorcarbonate; Zrn, zircon; Q, quartz; Xtm, xenotime-(Y); Th, thorite; Rt, rutile; Pl, plagioclase; Ttn, titanite; Mag, magnetite. Field of view: a, 500 μm; b, 700 μm; c, 150 μm and d, 700 μm.

Supplementary Table 1 | Trace element (ppm) compositions of drilling samples from Zhaibei area.

sample	MZS-316						MZS-510					MZS-512		
depth(m)	-2	-4	-6	-8	-10	-12	-2	-4	-6	-8	-10	-2	-4	-6
Rb	149	170	206	248	307	272	192	255	289	327	393	114	160	178
Sr	38.6	53.3	54.0	57.8	85.6	81.4	47.7	68.9	84.2	97.8	129	21.6	25.3	33.4
Y	172	202	49.4	29.3	20.0	18.0	106	71.2	36.2	22.2	36.0	78.8	121	148
Zr	212	195	190	148	174	150	188	209	153	175	188	248	226	170
Nb	37.3	36.4	35.8	36.6	38.3	30.5	25.0	24.5	23.8	32.7	22.2	43.4	39.4	34.2
Ba	180	237	233	311	369	456	176	285	350	426	496	74.3	90.6	155
La	299	192	59.2	45.2	35.7	32.9	151	89.8	41.5	29.5	52.4	174	263	233
Ce	68.2	86.5	33.7	138	42.9	33.1	95.8	62.0	72.3	57.0	59.7	136	87.6	71.0
Pr	55.1	35.6	10.8	8.68	7.34	6.41	32.6	17.0	8.13	5.61	10.2	33.9	54.0	45.6
Nd	195	126	35.1	28.3	23.6	21.0	114	60.6	28.5	21.1	36.5	120	169	145
Sm	40.5	28.6	7.83	5.72	4.30	4.22	23.7	12.6	5.80	4.15	7.03	21.4	31.5	30.2
Eu	10.1	7.68	1.92	1.28	1.02	0.99	5.27	2.69	1.37	0.92	1.58	4.07	6.45	6.58
Gd	32.3	28.3	6.75	4.73	3.26	3.0	17.4	9.38	4.80	3.23	5.24	17.8	19.7	24.5
Tb	6.20	5.95	1.46	0.90	0.69	0.60	3.34	2.06	0.91	0.68	1.0	2.78	4.02	5.09
Dy	32.8	32.7	8.39	5.08	3.71	3.30	17.8	11.3	5.21	3.49	5.79	13.1	21.0	27.0
Ho	7.35	7.28	1.84	1.16	0.88	0.73	4.05	2.63	1.28	0.79	1.26	2.88	4.75	6.0
Er	18.5	19.1	5.06	3.39	2.49	2.12	10.8	6.83	3.67	2.36	3.80	8.20	12.8	15.5
Tm	2.69	2.82	0.78	0.53	0.42	0.33	1.58	1.06	0.62	0.37	0.65	1.18	1.86	2.14
Yb	17.7	17.9	5.16	3.44	2.81	2.28	10.6	7.28	4.15	2.90	4.32	7.95	12.2	14.0
Lu	2.77	2.76	0.78	0.55	0.46	0.36	1.61	1.05	0.66	0.44	0.68	1.26	1.88	2.11
Hf	6.12	6.07	5.81	4.69	5.13	4.42	5.72	6.01	4.75	4.74	5.25	7.34	6.69	5.31
Ta	3.35	2.89	3.25	3.33	3.86	2.68	2.14	1.90	2.06	2.46	1.72	3.51	3.59	2.98
Pb	24.5	48.0	26.2	257	93.7	22.6	47.6	19.8	17.5	14.1	13.5	118	49.2	80.5
Th	26.6	31.1	22.0	28.8	25.5	21.3	34.4	28.0	29.2	31.3	30.6	38.9	26.6	21.8
U	9.78	11.0	9.24	8.27	13.8	12.1	5.81	8.33	8.04	11.8	9.59	4.19	4.01	3.25

Supplementary Table 1 | Continued.

sample	MZS-513					MZS-516									
depth(m)	-2	-4	-6	-8	-10	-2	-4	-6	-8	-10	-12	-14	-16	-18	-20
Rb	39.1	63.2	128	191	189	21.4	46.8	34.3	29.6	26.1	30.8	26.1	49.3	46.4	81.4
Sr	4.38	10.7	31.1	46.8	56.1	3.36	2.75	2.65	2.19	1.84	1.67	2.22	3.54	3.56	30.2
Y	16.9	67.6	141	172	136	8.84	9.07	11.8	13.1	9.72	11.6	35.3	259	347	159
Zr	227	251	231	285	221	137	112	221	164	177	128	77.6	175	132	142
Nb	42.4	41.6	33.3	40.4	34.7	15.8	17.1	17.5	16.0	12.1	20.5	1.38	27.5	11.8	3.17
Ba	34.2	63.5	168	297	246	38.8	70.3	56.1	57.3	61.5	62.3	64.4	78.0	107	136
La	66.7	238	334	278	232	15.7	17.1	20.4	22.5	25.1	49.2	106	181	111	61.1
Ce	69.7	80.7	83.3	30.8	40.6	93.6	70.0	60.0	82.4	54.9	64.2	68.4	90.3	102	72.7
Pr	13.2	48.1	62.4	52.4	43.2	3.64	5.40	6.01	7.50	9.58	17.8	28.1	41.2	25.2	15.2
Nd	48.5	168	225	182	152	13.7	21.8	23.8	30.9	41.1	76.1	114	168	105	63.0
Sm	7.70	29.2	42.2	38.4	32.5	2.82	4.71	5.22	6.05	6.41	11.7	23.7	45.6	29.2	14.8
Eu	1.42	5.88	9.54	9.29	7.53	0.73	1.08	1.31	1.43	1.24	2.17	6.03	16.5	12.8	5.23
Gd	4.2	16.0	27.0	29.8	23.5	2.93	3.59	4.20	4.90	4.40	6.76	15.8	52.1	46.3	16.8
Tb	0.74	2.94	5.13	5.78	5.0	0.42	0.58	0.66	0.76	0.65	0.90	2.37	9.08	9.71	3.98
Dy	3.3	13.9	25.6	29.1	25.5	2.32	2.83	3.54	3.96	3.07	3.89	9.97	49.6	60.7	23.8
Ho	0.70	3.0	5.70	6.60	5.70	0.49	0.58	0.72	0.81	0.64	0.82	2.08	10.6	13.6	5.29
Er	2.10	7.80	15.3	17.6	14.6	1.40	1.68	2.03	2.29	1.95	2.49	6.12	26.7	33.4	12.7
Tm	0.35	1.12	2.25	2.58	2.15	0.21	0.26	0.30	0.32	0.26	0.36	0.79	3.63	4.19	1.61
Yb	2.51	7.94	15.0	17.2	14.3	1.54	1.81	2.25	2.26	1.91	2.58	5.69	21.8	23.4	8.94
Lu	0.39	1.28	2.38	2.58	2.20	0.22	0.27	0.33	0.35	0.28	0.37	0.80	3.14	3.43	1.37
Hf	5.99	6.53	6.39	7.97	6.47	4.19	3.65	6.06	4.71	4.53	4.32	3.15	5.85	5.06	4.58
Ta	3.53	3.38	3.09	3.60	3.06	0.57	0.65	0.86	0.64	0.33	0.90	0.09	1.54	0.87	0.15
Pb	19.5	27.2	17.2	17.1	9.77	24.6	10.7	16.2	11.9	15.1	11.7	198	13.3	13.9	177
Th	22.6	26.8	24.6	30.5	28.9	7.87	8.57	13.0	6.50	9.72	5.58	4.45	6.16	7.75	8.39
U	5.51	5.02	5.02	6.46	6.09	1.65	1.50	1.92	1.72	2.13	1.15	0.86	1.52	1.73	1.84

Supplementary Table 1 | Continued.

sample	MZS-660			MZS-683				KZD-094			KZD-120			
depth(m)	-2	-4	-6	-2	-4	-6	-8	-2	-4	-6	-2	-4	-6	-8
Rb	455	295	357	279	310	299	279	245	236	280	210	231	230	189
Sr	17.0	18.7	15.5	102	136	173	212	37.5	45.5	49.3	37.2	50.3	48.1	34.6
Y	277	330	238	17.8	17.6	14.1	23.4	56.2	35.9	42.3	33.6	23.8	32.9	40.0
Zr	361	246	311	266	193	170	248	214	193	188	122	144	147	137
Nb	37.0	15.8	42.5	38.8	31.5	30.6	36.3	31.6	27.6	28.1	17.9	28.0	25.4	17.0
Ba	886	540	693	503	656	678	610	727	962	966	924	1350	1110	1050
La	295	246	168	34.2	32.9	26.1	35.8	74.3	57.9	65.2	69.7	44.3	58.3	55.4
Ce	213	75.6	390	45.8	96.1	59.6	44.5	114	74.7	141	99.9	96.5	59.3	346
Pr	67.6	52.8	35.2	6.61	6.33	4.97	6.80	18.5	12.5	16.4	15.9	10.0	12.9	12.6
Nd	239	191	131	23.1	22.7	17.6	24.9	65.2	45.8	55.3	56.0	36.0	48.2	46.3
Sm	54.7	49.2	30.9	4.35	4.14	3.41	4.79	13.9	9.6	11.2	10.5	6.64	8.97	8.59
Eu	9.09	8.16	5.42	0.99	1.08	0.84	1.21	2.07	1.52	1.76	2.10	1.57	1.93	1.71
Gd	44.7	47.6	33.0	3.14	3.50	2.59	3.45	10.4	6.17	8.82	7.35	4.70	6.23	8.23
Tb	10.0	10.8	7.48	0.60	0.58	0.48	0.69	2.25	1.39	1.61	1.42	0.92	1.22	1.36
Dy	55.3	60.8	42.5	3.20	3.22	2.54	3.66	11.3	7.30	8.36	7.31	4.55	6.53	7.54
Ho	11.1	12.2	9.20	0.74	0.70	0.59	0.85	2.44	1.53	1.76	1.53	0.95	1.41	1.74
Er	28.2	30.2	23.1	2.19	1.99	1.74	2.52	6.30	4.06	4.73	4.01	2.55	3.83	5.07
Tm	3.77	4.09	3.05	0.35	0.32	0.28	0.40	0.81	0.60	0.62	0.57	0.36	0.54	0.77
Yb	22.8	24.6	18.5	2.44	2.14	1.97	2.71	4.98	3.63	3.73	3.39	2.45	3.39	4.98
Lu	3.14	3.53	2.57	0.42	0.36	0.34	0.45	0.73	0.57	0.52	0.47	0.35	0.53	0.72
Hf	11.9	7.93	9.73	7.54	5.27	4.50	5.88	6.64	6.34	5.82	4.25	4.33	4.41	4.59
Ta	2.69	1.09	2.94	3.23	1.93	2.72	3.03	2.21	2.17	2.37	1.25	1.74	1.55	0.14
Pb	37.6	28.1	53.9	11.2	14.1	13.1	14.0	39.6	39.9	48.1	84.8	63.5	30.0	60.6
Th	58.6	37.8	50.9	23.5	25.7	22.8	23.6	30.9	29.8	32.6	25.0	27.6	26.2	22.2
U	18.6	9.66	25.6	9.51	8.30	8.45	10.7	5.40	3.98	4.06	4.68	6.15	6.37	7.56

Supplementary Table 1 | Continued.

sample	JZB-378						JZB-575			JZB-580						
depth(m)	-2	-4	-6	-8	-10	-12	-2	-4	-6	-2	-4	-6	-8	-10	-12	-14
Rb	142	199	214	173	204	250	187	278	254	219	279	309	269	416	408	436
Sr	6.90	10.7	12.2	8.10	11.6	15.1	11.1	25.8	30.2	70.8	75.6	99.8	89.3	117	114	156
Y	61.1	71.7	202	337	262	128	56.9	22.0	29.2	103	51.4	24.6	14.7	22.4	20.1	27.8
Zr	323	220	190	198	173	192	205	232	177	214	201	208	233	204	228	297
Nb	40.5	39.8	39.0	37.3	33.0	44.7	41.7	46.9	34.5	20.2	24.1	19.3	33.7	31.0	22.3	34.5
Ba	118	224	260	263	369	382	323	412	420	291	568	553	410	517	538	645
La	137	196	289	243	139	86.4	81.6	23.1	38.9	117	65.4	32.3	25.9	28.7	29.4	39.4
Ce	145	136	49.4	108	227	73.2	87.9	41.4	50.3	59.0	177	74.1	36.6	62.9	65.8	57.4
Pr	28.6	39.9	50.8	40.0	22.8	14.9	14.9	4.0	7.10	24.0	12.6	6.40	5.10	5.80	5.90	8.0
Nd	102	134	169	131	73.5	48.3	50.4	13.1	23.2	87.6	45.7	23.1	18.0	20.6	21.8	30.2
Sm	18.4	23.8	34.2	27.2	15.6	9.59	9.62	2.64	4.33	19.0	9.75	4.66	3.35	4.06	3.96	5.55
Eu	2.06	2.57	4.13	3.79	2.23	1.22	1.64	0.54	0.71	3.91	2.07	0.94	0.72	0.85	0.87	1.14
Gd	10.5	13.8	26.8	30.9	21.0	9.50	7.74	2.37	3.46	13.6	7.42	3.29	2.27	2.95	3.11	3.58
Tb	2.10	2.70	5.69	8.29	5.61	2.68	1.53	0.54	0.75	2.96	1.50	0.65	0.47	0.62	0.57	0.80
Dy	10.9	12.7	33.2	51.6	36.9	17.3	9.40	3.46	4.55	15.7	8.59	3.64	2.33	3.15	2.93	4.07
Ho	2.42	2.93	7.63	12.0	9.0	4.25	2.07	0.86	1.10	3.32	1.71	0.78	0.49	0.75	0.71	0.93
Er	6.98	8.29	20.7	31.9	23.9	11.4	5.99	2.52	3.39	9.33	5.15	2.40	1.45	2.24	2.10	2.85
Tm	1.13	1.19	2.98	4.67	3.50	1.74	0.96	0.46	0.55	1.37	0.80	0.40	0.24	0.36	0.31	0.47
Yb	7.93	8.80	19.6	29.7	22.3	11.4	6.67	3.33	3.87	9.62	5.50	2.86	1.69	2.84	2.53	3.33
Lu	1.20	1.28	2.75	4.19	3.09	1.70	0.97	0.53	0.63	1.38	0.82	0.46	0.25	0.44	0.38	0.55
Hf	8.75	5.95	5.77	6.03	5.06	5.74	6.03	6.71	4.99	5.30	5.71	5.64	5.88	5.84	6.23	8.36
Ta	3.55	3.29	3.97	3.41	3.11	4.34	4.80	5.60	3.74	1.54	1.70	1.45	2.62	2.40	1.66	2.49
Pb	21.7	15.2	12.4	10.3	17.2	13.8	21.5	14.9	16.6	16.9	43.9	26.5	15.0	18.7	39.9	35.7
Th	36.6	29.8	29.4	26.3	24.2	45.7	33.0	39.9	27.4	25.8	26.9	25.3	19.6	28.1	26.8	37.8
U	5.59	4.90	5.72	5.05	5.11	5.13	5.30	4.70	4.43	8.02	8.88	12.7	14.0	8.61	10.5	12.2

Supplementary Table 2 | LA-ICPMS U–Th–Pb age determinations of zircons from granites.

analysis #	1	2	3	4	5	6	7	8	9	10	11	12	13
Pb (ppm)	3.54	2.10	3.97	20.5	3.60	3.24	3.77	3.90	9.76	4.13	15.0	8.05	8.86
Th	57.0	38.0	69.1	266	52.8	40.7	69.0	59.6	182	69.0	283	64.4	157
U	88.9	55.5	111	572	97.4	87.6	97.9	111	250	107	392	239	238
Th/U	0.64	0.68	0.62	0.47	0.54	0.47	0.70	0.54	0.73	0.65	0.72	0.27	0.66
$^{207}Pb/^{206}Pb$	0.04967	0.05012	0.04994	0.05016	0.05006	0.05029	0.04943	0.04941	0.04978	0.04988	0.05011	0.05049	0.04994
1s	0.00619	0.00649	0.0051	0.00134	0.00499	0.00343	0.00535	0.00874	0.00163	0.00487	0.00196	0.00294	0.00214
$^{207}Pb/^{235}U$	0.21058	0.20426	0.19737	0.20255	0.20346	0.20537	0.20349	0.18601	0.20484	0.20775	0.20185	0.2042	0.19991
1s	0.02587	0.02584	0.0198	0.00526	0.02006	0.01382	0.02188	0.03236	0.00655	0.02013	0.0078	0.01161	0.00838
$^{206}Pb/^{238}U$	0.03076	0.02956	0.02867	0.0293	0.02948	0.02962	0.02987	0.02731	0.02985	0.03022	0.02923	0.02934	0.02904
1s	0.00077	0.00092	0.00067	0.00035	0.00055	0.00047	0.00049	0.00098	0.00037	0.00048	0.00035	0.00049	0.00041
$^{207}Pb/^{235}U$ *age (Ma)*	194	189	183	187	188	190	188	173	189	192	187	189	185
1s	22	22	17	4	17	12	18	28	6	17	7	10	7
$^{206}Pb/^{238}U$ *age (Ma)*	195	188	182	186	187	188	190	174	190	192	186	186	185
1s	5	6	4	2	3	3	3	6	2	3	2	3	3

Supplementary Table 2 | Continued.

analysis #	14	15	6	17	18	19	20	21	22	23	24	25	26
Pb	6.73	6.57	6.71	5.71	2.87	15.5	5.80	3.63	3.11	9.37	4.48	6.28	2.60
Th	136	109	102	96.7	62.2	242	104	42.2	41.2	132	75.7	70.6	34.2
U	175	175	172	150	74.6	425	150	102	91.1	245	119	173	67.7
Th/U	0.78	0.62	0.60	0.64	0.83	0.57	0.70	0.41	0.45	0.54	0.64	0.41	0.51
$^{207}Pb/^{206}Pb$	0.05008	0.04941	0.04955	0.04968	0.04959	0.04992	0.04962	0.04956	0.04928	0.05022	0.05007	0.04992	0.0497
1s	0.0022	0.00259	0.00336	0.00474	0.00395	0.0015	0.005	0.00326	0.00522	0.00299	0.00273	0.0017	0.0038
$^{207}Pb/^{235}U$	0.1997	0.199	0.20861	0.20043	0.19516	0.1997	0.20421	0.20265	0.18811	0.21263	0.20436	0.20746	0.21328
1s	0.00863	0.01017	0.01378	0.01863	0.01535	0.00591	0.02035	0.01319	0.01975	0.01258	0.01091	0.00693	0.0161
$^{206}Pb/^{238}U$	0.02893	0.02922	0.03054	0.02927	0.02855	0.02902	0.02986	0.02967	0.02769	0.03072	0.02961	0.03015	0.03113
1s	0.00037	0.00047	0.00058	0.00073	0.00049	0.00032	0.00058	0.00043	0.0005	0.00039	0.00046	0.00037	0.00051
$^{207}Pb/^{235}U$ age (Ma)	185	184	192	185	181	185	189	187	175	196	189	191	196
1s	7	9	12	16	13	5	17	11	17	11	9	6	13
$^{206}Pb/^{238}U$ age (Ma)	184	186	194	186	181	184	190	188	176	195	188	191	198
1s	2	3	4	5	3	2	4	3	3	2	3	2	3

Supplementary Table 3 | Compositions (wt.%) of minerals in granites.

sample	K-feldspar(20)[a]	plagioclase(17)	biotite(15)	zircon(28)	thorite(6)	titanite(3)	magnetite(3)	ilmenite(4)	calcite(5)
SiO_2	64.0(52)[b]	65.71(191)	35.71(85)	31.93(46)	17.88(82)	29.58(10)	0.08(3)		0.05(4)
TiO_2	bdl	bdl	3.19(71)		0.05(4)	34.58(40)	0.03(3)	49.70(13)	
Al_2O_3	18.34(30)	21.58(134)	14.96(277)		0.33(26)	1.40(16)	0.07(4)		
FeO	0.20(24)	0.16(13)	27.51(230)	0.30(39)	1.79(143)	0.71(13)	93.18(60)	36.98(124)	0.08(4)
MnO	bdl	bdl	0.36(26)		0.07(5)	bdl	0.12(6)	9.69(129)	0.43(30)
MgO	bdl	bdl	4.50(87)		0.16(32)	0.02(1)	bdl		0.01(1)
CaO	0.03(8)	2.18(143)	0.05(11)	0.03(5)	1.90(40)	26.72(13)	bdl		53.45(18)
BaO	0.24(27)	bdl							
Na_2O	0.72(83)	9.97(105)			0.02(2)	0.03(2)	0.13(13)		
K_2O	15.38(131)	0.34(45)	9.01(48)		bdl	0.01(1)	0.01(2)		
P_2O_5									0.03(2)
SrO									1.78(6)
Nb_2O_5						0.32(13)		1.80(13)	
Ta_2O_5						0.01(1)		0.16(10)	
ZrO_2				64.39(121)		1.76(55)			
HfO_2				1.46(29)					
ThO_2				0.30(22)	57.0(369)	0.06(9)			
UO_2				0.23(21)	9.46(407)	0.01(1)			
Y_2O_3				0.46(30)	0.54(33)				
Dy_2O_3				0.04(3)					
Er_2O_3				0.09(5)					
Yb_2O_3				0.14(10)					
F			0.68(47)		0.65(18)	0.46(9)			
CO_2[c]									43.19(36)
H_2O[d]			3.92(35)						
O=F			-0.29(20)		-0.27(8)	-0.19(4)			
Total	98.91(65)	99.95(38)	99.31(130)	99.41(61)	89.59(159)	95.64(42)	93.67(54)	98.31(30)	99.09(89)

Supplementary Table 3 | Continued REE-rich minerals.

sample	fluorapatite (14)	monazite-(Ce) (10)	xenotime-(Y) (2)	fluorcarbonate-(Ce) (23)	REE-1 (15)	REE-2 (8)	REE-3 (34)
SiO_2	0.37(56)	1.05(53)	3.34(56)	0.68(84)	0.09(4)	0.41(57)	0.23(56)
P_2O_5	39.97(93)	29.20(185)	29.67(239)	0.02(2)	24.79(146)	22.76(158)	0.15(20)
CaO	54.07(148)	1.39(54)	0.10(7)	15.15(341)	0.31(37)	0.74(179)	0.48(32)
Y_2O_3	0.33(35)	0.74(23)	46.0(620)	1.96(171)	17.24(730)	45.54(531)	6.75(380)
La_2O_3	0.11(13)	11.37(99)	0.01(2)	11.61(88)	9.26(208)	0.67(69)	29.07(223)
Ce_2O_3	0.34(33)	25.25(168)	0.03(4)	22.80(282)	0.21(21)	0.03(6)	0.07(17)
Pr_2O_3	0.05(7)	6.83(67)	bdl	2.04(18)	2.55(115)	0.23(20)	9.11(397)
Nd_2O_3	0.20(20)	10.53(92)	0.06(4)	6.10(63)	11.48(611)	1.52(110)	18.58(269)
Sm_2O_3	0.09(11)	1.94(26)	0.13(4)	1.49(135)	4.07(294)	1.03(118)	4.27(42)
Eu_2O_3			0.01(1)	0.06(5)	0.13(23)	bdl	0.37(37)
Gd_2O_3			0.59(25)	0.97(61)	3.14(59)	1.63(41)	2.02(155)
Dy_2O_3			2.96(182)	0.39(22)	7.34(151)	8.58(39)	1.42(111)
Er_2O_3			4.11(204)	0.15(11)	1.28(54)	4.18(53)	0.51(31)
Yb_2O_3			6.15(199)		0.77(38)	2.87(54)	
ThO_2	0.01(2)	8.48(339)	0.09(12)	1.54(153)	0.02(30)	0.02(3)	0.07(9)
UO_2	bdl	0.97(55)	0.45(33)	0.04(4)	bdl	bdl	0.03(3)
F	3.74(70)	0.82(19)	0.75(36)	5.10(80)	1.01(30)	7.55(108)	5.46(268)
CO_2[c]				26.51(102)			21.48(96)
H_2O[d]				1.08(86)			2.40(183)
O=F	-1.58(29)	-0.35(8)	-0.32(15)	-2.15(34)	-0.42(13)	-3.18(45)	-2.30(113)
Total	97.68(99)	98.21(150)	94.14(209)	95.53(576)	83.69(585)	94.57(458)	99.90(375)

[a]EMP analyses number.
[b]one standard deviation in the rightmost digit.
[c]calculated using charge balance method.
[d]calculated assuming a full (F,OH) site.
bdl, below determined limits of microprobe.
H_2O in REE-1 cannot be well calculated because of unknown chemical structure, and in REE-3 is calculated as possibly bastnäsite-(La).

Supplementary Table 4 | Major (wt%) and trace (ppm) compositions of granites and fluorapatite and secondary REE minerals.

sample	MZS-a	MZS-b	MZS-c	MZS-d	JZB-a	JZB-b	KZD-a	KZD-b	KZD-c
SiO_2	72.47	73.96	69.30	78.0	68.03	68.45	71.86	71.84	73.14
TiO_2	0.23	0.17	0.36	0.14	0.35	0.30	0.48	0.41	0.34
Al_2O_3	14.19	13.48	16.08	11.49	16.4	14.03	13.33	13.22	13.09
FeO	2.20	2.45	2.67	1.74	2.83	2.40	3.98	4.28	3.28
MnO	0.01	0.01	0.04	0.01	0.09	0.08	0.03	0.06	0.02
MgO	0.19	0.19	0.34	0.08	0.47	0.34	0.33	0.26	0.26
CaO	0.43	0.20	1.38	0.20	1.53	2.71	0.78	0.05	0.60
Na_2O	4.27	3.98	3.95	1.79	4.12	4.28	2.34	1.81	2.10
K_2O	4.45	4.24	4.39	5.62	4.44	4.41	4.83	5.01	5.27
P_2O_5	0.03	0.03	0.07	0.01	0.06	0.06	0.04	0.04	0.06
LOI	0.87	1.09	1.16	0.83	1.36	2.58	1.41	2.43	1.56
Total	99.36	99.81	99.82	99.91	99.78	99.72	99.49	99.50	99.81
Rb	273	266	244	357	260	280	263	214	299
Sr	63.4	49.5	404	129	419	357	80.8	64.1	77.7
Y	588	118	62.5	36.8	24.0	18.9	161	33.7	103
Zr	156	207	163	178	181	103	140	152	113
Nb	33.0	41.9	31.6	20.9	32.2	23.3	25.3	24.3	23.0
Ba	236	253	542	81.6	582	489	898	985	916
La	1238	129	90.8	74.9	35.6	24.0	127	50.1	81.8
Ce	56.9	86.3	67.1	96.5	58.2	43.8	102	59.4	68.3
Pr	245	24.7	18.8	18.8	7.60	5.0	40.7	12.0	17.4
Nd	743	83.0	69.1	68.3	27.8	18.4	147	43.4	59.3
Sm	178	17.1	13.6	13.8	5.0	3.50	32.3	9.10	12.4
Eu	22.9	1.72	3.39	1.71	1.32	0.92	3.91	1.43	2.02
Gd	158	17.5	13.5	12.2	4.80	3.72	26.6	7.69	12.6
Tb	25.6	2.82	1.99	1.66	0.70	0.51	5.25	1.26	2.75
Dy	125	16.4	10.6	8.15	3.64	2.90	29.9	6.66	16.7
Ho	23.0	3.90	2.29	1.71	0.85	0.65	5.93	1.43	3.64
Er	55.7	11.1	6.21	4.40	2.38	1.80	14.4	3.58	8.78
Tm	7.59	1.71	0.93	0.62	0.37	0.30	1.81	0.53	1.16
Yb	48.7	11.3	6.32	4.11	2.75	2.17	10.8	3.26	6.92
Lu	6.02	1.64	0.91	0.61	0.44	0.33	1.39	0.46	0.92
Hf	6.56	6.77	4.24	7.46	4.52	2.86	4.60	4.49	4.01
Ta	4.38	4.40	2.57	1.12	2.38	1.65	1.77	1.63	1.63
Pb	10.5	10.1	10.6	20.9	12.4	19.0	33.1	25.6	33.1
Th	29.4	36.8	23.7	37.0	27.4	29.3	25.6	26.1	28.9
U	7.22	6.84	7.66	10.2	6.24	5.52	5.77	5.89	6.57

Supplementary Table 4 | Continued fluorapatite(Ap), REE-1 and REE-3.

sample	Ap(5)[a]	REE-1(4)	REE-3(4)
Rb	1.74(325)		
Sr	64.4(79)		
Y	2908(234)	111400(44092)	95832(1270)
Zr	5.06(781)		
Nb	6.99(1552)		
Ba	37.1(656)		
La	361(52)	76742	255805
Ce	1222(186)	2959(1612)	446(48)
Pr	201(31)	21843(4680)	38676(297)
Nd	1070(156)	80940(17637)	129309(1316)
Sm	446(50)	24086(4214)	31067(257)
Eu	7.11(102)	3332(451)	4330(54)
Gd	537(52)	23276(3402)	29035(239)
Tb	104(9)	4803(1278)	4922(33)
Dy	611(54)	28427(8601)	24713(119)
Ho	107(9)	4735(1471)	3693(26)
Er	229(20)	11837(3449)	7762(35)
Tm	23.4(23)	1786(473)	938(8)
Yb	120(14)	13616(3352)	5884(77)
Lu	13.5(17)	1496(386)	578(12)
Hf	0.08(14)	2.47(142)	2.30(37)
Ta	0.31(67)		
Pb	22.1(73)	96.5(381)	48.9(42)
Th	47.3(313)	35.5(130)	14.1(49)
U	114(25)	945(215)	66.1(161)
La/Yb_{CN}[b]	2.1(4)	4.1(13)	29.5(4)
δCe[c]	1.10(1)	0.02(1)	0.001(0)
δEu[d]	0.04(1)	0.43(5)	0.439(4)

[a]in-situ LA-ICP-MS analyses number.
[b]Chondrite normalized (CN) ratio.
[c]$Ce_{CN}/(La_{CN}*Pr_{CN})^{1/2}$.
[d]$Eu_{CN}/(Sm_{CN}*Gd_{CN})^{1/2}$.

Supplementary Table 5 | Sr, Nd isotopes of feldspar, Sr-rich calcite and REE-rich minerals in granites.

sample	feldspar			calcite									
analysis #	1	2	3	1	2	3	4	5	6	7	8	9	10
Rb[a]	180	70.3	117										
Sr	149	90.1	116										
$^{87}Rb/^{86}Sr$	3.445	2.228	2.882	<0.0001	<0.0001	0.0001	0.0008	0.0001	<0.0001	<0.0001	0.0009	0.0014	0.0018
$^{87}Sr/^{86}Sr$	0.718008	0.717260	0.731820	0.70611	0.70611	0.70563	0.70536	0.70609	0.70602	0.70608	0.70609	0.70601	0.70599
±2s	20	9	17	3	4	17	42	10	5	2	7	6	9
$(^{87}Sr/^{86}Sr)$)i[b]	0.70880	0.71130	0.72412	0.70611	0.70611	0.70563	0.70536	0.70609	0.70602	0.70608	0.70609	0.70601	0.70599
Sm	1.09	0.49	0.50										
Nd	6.70	2.83	3.50										
$^{147}Sm/^{144}Nd$	0.098	0.105	0.086										
$^{143}Nd/^{144}Nd$	0.511843	0.512424	0.511953										
±2s	18	20	17										
$(^{143}Nd/^{144}Nd)i$[b]	0.511722	0.512296	0.511847										
εNd(t)[c]	-13.4	-2.2	-10.9										

Supplementary Table 5 | Continued Nd isotope of REE-rich minerals.

sample	fluorapatite		monazite-(Ce)								REE-3		
analysis #	1	2	1	2	3	4	5	6	7	8	1	2	3
$^{147}Sm/^{144}Nd$	0.24457	0.24973	0.12504	0.12158	0.12204	0.11937	0.11686	0.11336	0.11248	0.11865	0.13639	0.13423	0.13030
±2s	14	18	15	5	26	18	17	12	13	35	7	8	11
$^{143}Nd/^{144}Nd$	0.512138	0.512175	0.511949	0.511928	0.511935	0.511944	0.511949	0.511962	0.511937	0.511934	0.512677	0.512657	0.512664
±2s	53	77	21	30	23	20	31	23	26	26	35	42	37
$(^{143}Nd/^{144}Nd)_i$[b]	0.511837	0.511867	0.511795	0.511778	0.511785	0.511797	0.511805	0.511823	0.511799	0.511788	0.512509	0.512492	0.512503
$\varepsilon_{Nd}(t)$[c]	-10.9	-10.3	-11.7	-12.1	-11.9	-11.7	-11.5	-11.2	-11.7	-11.9	2.2	1.9	2.1
sample	REE-3							REE-1					
analysis #	4	5	6	7	8	9	10	1	2	3	4	5	
$^{147}Sm/^{144}Nd$	0.15928	0.15889	0.15865	0.15945	0.15794	0.16066	0.15899	0.20043	0.20332	0.19096	0.18107	0.19525	
±2s	7	11	14	9	11	19	15	22	13	66	53	69	
$^{143}Nd/^{144}Nd$	0.512650	0.512632	0.512642	0.512645	0.512645	0.512646	0.512646	0.512564	0.512660	0.512661	0.512580	0.512619	
±2s	17	22	23	21	19	21	20	35	31	43	85	23	
$(^{143}Nd/^{144}Nd)i$	0.512454	0.512437	0.512447	0.512448	0.512450	0.512449	0.512451	0.512317	0.512410	0.512426	0.512358	0.512379	
$\varepsilon Nd(t)$	1.1	0.8	1.0	1.0	1.1	1.0	1.1	-1.5	0.3	0.6	-0.7	-0.3	

[a]Trace elements were analysed by solution ICPMS.

[b]Initial Sr, Nd isotope ratios are calculated assuming an age of 188 Ma.

[c]$\varepsilon_{Nd}(t)$ values are calculated based on present-day $(^{147}Sm/^{143}Nd)CHUR = 0.1967$ and $(^{143}Nd/^{144}Nd)CHUR = 0.512638$. Sr, Nd isotopes of feldspar were measured by solution MC-ICPMS, and Sr isotope of calcite, Nd of REE-rich minerals by in-situ LA-MC-ICPMS.
